# Chemical composition and bioactivity of essential oils and Ethanolic extracts of *Ocimum basilicum* L. and *Thymus algeriensis* Boiss. & Reut*.* from the Algerian Saharan Atlas

**DOI:** 10.1186/s12906-019-2556-y

**Published:** 2019-06-21

**Authors:** Maria Rezzoug, Boulanouar Bakchiche, Abdelaziz Gherib, Ascrizzi Roberta, FlaminiGuido, Özge Kilinçarslan, Ramazan Mammadov, Sanaa K. Bardaweel

**Affiliations:** 1 Laboratory of Process Engineering, Faculty of Technology, Laghouat University, 03000 Laghouat, Algeria; 2 Dipartimento di Farmacia, Università di Pisa, Via Bonanno 6, 56126 Pisa, Italy; 3 Department of Biology, Faculty of Science and Arts, Pamukkale University, Denizli, Turkey; 4 Department of Pharmaceutical Sciences, School of Pharmacy, The University of Jordan, Queen Rania Street, Amman, 11942 Jordan

**Keywords:** *Ocimum basilicum*, *Thymus algeriensis*, Essential oil, Ethanolic extract, Antioxidant activity, Antimicrobial activity, Anticancer activity

## Abstract

**Background:**

There is increasing interest in the pharmaceutical and food industries to substitute synthetic chemicals with naturally occurring compounds possessing bioactive properties. Plants are valuable sources of bioactive compounds. The present study investigates the chemical composition and antioxidant, antimicrobial, and anticancer activities of ethanolic extracts (EEs) and essential oils (EOs) from two species in the Lamiaceae family, *Ocimum basilicum* L. and *Thymus algeriensis* Boiss. & Reut., cultivated in the Algerian Saharan Atlas.

**Methods:**

The total flavonoid contents of the plants’ ethanolic extracts were determined by the aluminium chloride method, while the total phenols were determined using the Folin-Ciocalteu method. Essential oils were obtained by hydrodistillation of the aerial parts of the plants and were analysed by GC-MS. The free radical-scavenging ability and antioxidant potential of the plants’ EEs and EOs were probed using the 2, 2-diphenyl-picrylhydrazyl radical-scavenging, ABTS radical-scavenging, ferric-reducing power and phosphomolybdenum assays. The antimicrobial activities were evaluated against several pathogens characteristic of gram-negative bacteria (three species), gram-positive bacteria (three species) and fungi (two species). The microdilution method was used to estimate the minimum inhibitory concentrations (MICs). The oils’ anticancer potential against several cancer types was also studied using the MTT assay and reported as the toxic doses that resulted in a 50% reduction in cancer cell growth (LD_50_).

**Results:**

Phenolic compounds in the EEs from both plants were analysed by HPLC and demonstrated a rich flavonoid content. Chemical analysis of the essential oil from *Ocimum basilicum* revealed 26 unique compounds, with linalool (52.1%) and linalyl acetate (19.1%) as the major compounds. A total of 29 compounds were identified in the essential oil from *Thymus algeriensis*, with α-terpinyl acetate (47.4%), neryl acetate (9.6%), and α-pinene (6.8%) as the major compounds. The ethanolic extracts and essential oils from both plants exhibited moderate antioxidant activities and moderate to weak antimicrobial activities. Furthermore, anticancer activities against the examined human cancer cell lines were associated with only the EOs from both plants, with LD_50_ values ranging between 300 and 1000 μg/mL.

**Conclusion:**

The results suggest that the bioactive compounds found in the ethanolic extracts and essential oils from *Ocimum basilicum* and *Thymus algeriensis*, with diverse antioxidant, antimicrobial and anticancer activities, may have beneficial applications in nutraceutical and pharmaceutical technologies.

## Background

Among the numerous medicinal plants, some prevalent species are particularly popular in folk medicine due to their antioxidant properties and health benefits [1]. The Lamiaceae family is one of the most extensively studied plant families containing members with proven human health benefits. Antimicrobial [2–4], antioxidant [5, 6], anti-inflammatory, anti-depressive, and antiproliferative effects [7, 8] have been demonstrated for the active compounds found in many plants from this family. Rosemary, thyme, basil, and sage are famous members of the Lamiaceae family and are among the most commonly used plants in traditional Mediterranean remedies, mainly for the treatment of gastritis, infections, dermatitis, bronchitis, and inflammation [3, 4, 9, 10].

*Ocimum basilicum,* an aromatic herb that belongs to the Lamiaceae family and the Nepetoideae subfamily [9], has been used in folk medicine for the treatment of gastrointestinal and respiratory diseases [10]. This species was also reported as having beneficial effects on kidney malfunctions, warts and worm infestations [10]. Various chemical functionalities characteristic of anthocyanins, flavonoids, monoterpenes, phenolic acids and their esters, phenylpropanoid derivatives, phytosterols, tannins, and triterpenes have been identified in different *O. basilicum* extracts [11, 12]. Essential oils extracted from its leaves and flowers can be used as flavouring agents in food, medicine and cosmetics [10, 11].

Another renowned genus of the Lamiaceae family is the *Thymus* genus. It contains approximately 215 to 350 species that are remarkably predominant in the Mediterranean area [13, 14]. The aerial parts and the volatile components of *Thymus* species are traditionally used as herbal teas and condiments and for numerous medicinal purposes, e.g., as antispasmodic, antibacterial, antiviral, expectorant and antioxidant agents [15]. Compounds in several classes have been identified in *Thymus* species, such as carvacrol, thymol, geraniol, γ-terpineol, thujone and linalool [16].

The aim of this study was to investigate the chemical composition and biological activities of essential oils and ethanolic extracts from *Ocimum basilicum* and *Thymus algeriensis* cultivated in the Algerian Saharan Atlas (Laghouat region).

## Materials and methods

### Plant materials

*Ocimum basilicum* and *Thymus algeriensis* were harvested in their flowering stage between April and May 2016 in the area of the Algerian Saharan Atlas (Laghouat region). The species were characterized at the Department of Biology, Faculty of Science, University of Laghouat (Algeria), and the voucher specimens were banked at the Laboratory of Process Engineering, University of Laghouat, with the numbers LGP Ob/04/16 and LGP Ta/05/16, respectively. The plant materials were air-dried for 15 days and stored at room temperature (25 ± 2 °C) without exposure to direct sunlight.

### Preparation of the ethanolic extracts

Dried plant aerial parts (leaves) were pulverized. Each 15 g of ground sample was placed into a separate Erlenmeyer flask. Then, 100 mL of ethanol (100%) was added, and the samples were incubated in a water bath at 55 °C for 6 h. Separation of the extraction mixture from the residue was achieved by filtration through Whatman No. 1 filter paper. Each plant residue was re-extracted in triplicate with ethanol. After filtration, the two portions were mixed. The residual solvent in the ethanolic extracts were removed under reduced pressure at 48–49 °C using a rotary evaporator (Rotavapor IKA VB 10, Germany). Water in the extracts was lyophilized using a freeze dryer (Thermo Savant Modulyo D, USA) for 8 h at − 50 °C and 0.040 mbar. The yields of these fractions were 20.16% (*Ocimum basilicum*) and 15.78% (*Thymus algeriensis*).

### Total phenolic content

The total phenolic content was determined with Folin-Ciocalteu reagent using the method described by Singleton et al. [17]. Briefly, to 200 μL of each sample (three replicates), 1 mL of 10% (v/v) Folin-Ciocalteu reagent and 800 μL of Na_2_CO_3_ (7.5%, w/v) were added and incubated at 25 °C for 30 min. The absorbance of each sample was measured at 760 nm using a spectrophotometer (OPTIZEN 2120 UV). An ethanol solution of gallic acid was tested in parallel to obtain a calibration curve. Values were expressed as milligrams of gallic acid equivalents per gram of dry weight (mg GAE/g dry weight).

### Total flavonoid content

The total flavonoid content was estimated using the aluminium chloride colorimetric method of Boulanouar et al. [18]. Briefly, 1 mL of diluted extract was mixed with 1 mL of a 2% AlCl_3_ methanol solution. After incubation at room temperature for 15 min, the absorbance was measured at 430 nm using a spectrophotometer (OPTIZEN 2120 UV). The total flavonoid content was calculated from the calibration curve of rutin and expressed as milligrams of rutin equivalents per gram of dry weight (mg RE/g dry weight).

### Analysis of phenolic contents by HPLC

The HPLC procedure described by Caponio et al. [19] was adopted for the analysis of phenolic contents. The conditions utilized were as follows: a C-18 column (CTO-10ASVp, 4.6 mm × 250 mm, 5 μM), an injection volume of 20 μL, a mobile phase composed of solvent A (3% formic acid in distilled water) and solvent B (100% acetonitrile), gradient elution from 15 to 100% B over a run time of 45 min at a flow rate of 1 mL/min, and a UV-Vis detector. For analysis, the samples were dissolved in ethanol, and 20 μL of this solution was injected into the column. Chromatograms were plotted based on detection at 280 nm with the UV-Vis detector. The retention times and UV absorption spectra of the phenolic compounds were compared with those of pure standards. Gallic acid, 3, 4-dihydroxybenzoic acid, 4-hydroxybenzoic acid, 2, 5-dihydroxybenzoic acid, chlorogenic acid, vanillic acid, caffeic acid, p-coumaric acid, ferulic acid, rutin, ellagic acid, naringin, and quercetin were used as standards. Peaks were identified by matching the retention times and UV spectra with those of the standards. The quantity of each phenolic compound was reported in μg per gram of the extract.

### Isolation of essential oils

Essential oils were extracted from the air-dried and ground plants (100 g each) using the hydrodistillation method for 4 h in a Clevenger-type apparatus. The obtained essential oils were dried over anhydrous sodium sulfate and stored at 4 °C in the dark until further analysis.

### GC-MS analysis

GC-EIMS analyses were accomplished with a Varian CP-3800 gas chromatograph equipped with a DB-5 capillary column (30 m × 0.25 mm; coating thickness of 0.25 μm) and a Varian Saturn 2000 ion trap mass detector. The following analytical specifications were used: injector and transfer line temperatures of 220 °C and 240 °C, respectively; oven temperature programmed from 60 °C to 240 °C at 3 °C/min; carrier gas of helium at 1 mL/min; injection volume of 0.2 μL (10% hexane solution); and split ratio of 1:30. For the identification of each compound, a comparison of the retention times with the standards’ retention times was performed. In addition, we evaluated the linear retention indices in relation to the retention times of a series of *n*-hydrocarbons and used computer-aided matching with commercially available mass spectra and mass spectra contained in an in-house library.

### Antioxidant activities

#### Free radical-scavenging activity

The DPPH radical-scavenging capacity was measured as previously described by Boulanouar et al. [18]. In this study, 1 mL of either ethanolic extract or essential oil was added to 2 mL of a 60 μM DPPH methanol solution, and the reaction mixture was kept at room temperature and away from light for 30 min before the absorbance was recorded at 517 nm. The following equation was used to estimate the scavenging activity: scavenging effect (%) = [100*(A_c_ - A_s_/ A_c_)], where A_c_ is the control sample absorbance and A_s_ is the test sample absorbance. The concentration of oil or extract at which 50% of the DPPH radicals (IC_50_) were scavenged was calculated. Ascorbic acid was employed as a reference compound.

#### ABTS assay

The antioxidant capacity was estimated following the procedure described by Re et al. [20] with minor modifications. In brief, a 7 mM ABTS stock solution was reacted with 2.45 mM potassium persulfate at room temperature for 12–16 h while protected from light to produce the ABTS radical cation (ABTS^•+^). The ABTS^•+^ mixture (1450 μL) was diluted with methanol to reach an absorbance of 0.70 ± 0.02 at 734 nm, followed by the addition of 50 μL of either the test sample or Trolox standard and incubation at room temperature while protected from light for 6 min. The absorbance was read at 734 nm using a spectrophotometer (OPTIZEN 2120 UV). For each test sample, the percent inhibition of ABTS^•+^ was computed using the following formula: (% inhibition) = [100*(A_c_ - A_s_ / A_c_)], where A_c_ is the control sample absorbance and A_s_ is the test sample absorbance. The concentration of oil or extract that could scavenge 50% of the ABTS radicals (IC_50_) was calculated. Trolox was used as a reference compound.

#### Reducing power assay

The method of Oyaizu 1986 was adopted to determine the reducing power of the examined EEs and EOs [21]. Extract solution (0.1 mL), phosphate buffer (2 mL, 0.2 M, pH 6.6) and potassium ferricyanide [K_3_Fe(CN)_6_] (2 mL, 1%) were mixed and then incubated at 50 °C for 20 min. Afterward, 2 mL of trichloroacetic acid (10%) was added to the solution to terminate the reaction. The mixture was then centrifuged at 3000 rpm for 10 min, and the upper layer of the solution (2 mL) was collected. The collected layer was mixed with distilled water (2.5 mL) and FeCl_3_ (0.5 mL, 0.1%) before the absorbance was measured at 700 nm. Greater reducing power was associated with increased absorbance of the reaction mixture. The results are expressed in terms of the ascorbic acid equivalent antioxidant capacity (AEAC).

#### Phosphomolybdenum assay

The Prieto et al. method was adopted to evaluate the reducing capacity of the EEs and EOs from the two assessed plants [22]. In summary, 0.3 mL of each diluted extract was added to 3 mL of a phosphomolybdic reagent solution containing ammonium molybdate (4 M), sodium phosphate (28 mM) and sulfuric acid (0.6 M). The mixture was placed in a water bath at a temperature of 95 °C for 90 min. After cooling to room temperature, the absorbance was measured at 695 nm against a blank solution containing 0.3 mL of ethanol mixed with 3 mL of phosphomolybdic reagent. The results are expressed in terms of the ascorbic acid equivalent antioxidant capacity (AEAC).

### Antimicrobial activity

#### Microorganisms

Six bacterial and two fungal species were acquired from the Microbial Culture Collection Center of the Medical School at the University of Jordan, Jordan. The species used were *Staphylococcus epidermidis* ATCC12228 (gram-positive bacterium), *Staphylococcus aureus* ATCC25923 (gram-positive bacterium), *Bacillus subtilis* ATCC11562 (gram-positive bacterium), *Escherichia coli* ATCC29425 (gram-negative bacterium), *Pseudomonas aeruginosa* ATCC15442 (gram-negative bacterium), *Klebsiella pneumoniae* ATCC43816 (gram-negative bacterium), *Candida glabrata* ATCC22553 (fungus), and *Candida albicans* ATCC10231 (fungus). The eight microorganisms represent predominant food pathogens that are frequently encountered [23–25].

#### MIC determination

Measurements of the minimum inhibitory concentration (MIC), defined as the minimum concentration at which more than 80% of the microbial growth is restrained, were performed in 96 flat-bottom microtiter plates (TPP, Switzerland) in accordance with the microdilution method, as previously reported by Bardaweel et al. [23]. An inoculum volume of 1 × 10^5^ CFU mL^− 1^ for each microorganism was used in each microtiter plate well. Ampicillin and amphotericin B (positive controls), as well as media (negative control), were employed under comparable experimental conditions. Plates containing bacteria were placed in a shaker incubator for 48 h at 37 °C, whereas plates containing *Candida* were incubated in the shaker for 48 h at 33 °C. To assess microbial growth, optical densities were read at 600 nm (OD_600_) using a microplate reader (Palo Alto, CA, USA).

### Anticancer activity

#### Cell lines and cell viability

All cancer cell lines used in this study (MCF7, MDA-MB-231 HeLa, PC3, and K562) were acquired from the American Type Culture Collection (Rockville, MD, USA). All cells were cultured in Dulbecco’s modified Eagle’s medium (DMEM) supplemented with 10% foetal bovine serum, 100 U/mL penicillin, and 100 μg/mL streptomycin at 37 °C with 5% CO_2_. The count of viable cells was determined using the trypan blue method [24].

#### MTT assay

The anticancer activity of the phenolic extracts and essential oils were studied in 96-well round-bottom microplates using the MTT (3-[4, 5-dimethylthiazole-2-yl]-2, 5-diphenyl-tetrazolium bromide) assay (Sigma-Aldrich, USA) as previously described by Bouziane et al. [25]. In summary, cells were seeded in 96-well plates at a cell density of 1 × 10^4^ cells/mL and incubated for 24 h to permit attachment. Each well was treated with different concentrations (0.001–1000 μg/mL) of the prepared extracts in triplicate and incubated for 48 h. Subsequently, 10 μL of 0.5 mg/mL MTT was added to each well and further incubated for 4 h before measuring the absorbance at 570 nm. Growth inhibition was determined based on the following equation: inhibition (%) = 100-(mean Abs of test sample - mean Abs of negative control) ×100**/**(mean Abs of positive control-mean Abs of negative control). Graph Pad Prism 7 software (GraphPad Software, Inc., La Jolla, CA, USA) was used for data analysis to determine the inhibition percentage, and the results are presented as the LD_50_ value, described as the concentration that produces 50% growth suppression. The positive control doxorubicin was employed under the same experimental conditions.

### Data analysis

For all experiments, the analyses were performed in triplicate, and the values are reported as the mean ± standard deviation (SD). The results were analysed via Student’s t-test, with α = 0.05. The analyses were carried out using IBM SPSS Statistics for Windows, version 22.0. (IBM Corp., Armonk, New York, USA).

## Results and discussion

### Total phenolic and flavonoid contents

The total phenol contents of extracts estimated by the Folin-Ciocalteu procedure and the total flavonoid contents estimated by the AlCl_3_ method are presented in Table 1. The ethanolic extract of *O. basilicum* had the highest phenolic compound content and flavonoid content (226 mg/g, gallic acid equivalents, and 213 mg/g, rutin equivalents).

**Table 1:** Total phenolic and flavonoids contents in the ethanolic extracts of *Ocimum basilicum* and *Thymus algeriensis*

Plant	phenolic content (mg/g extract)	flavonoid content (mg/g extract)
*Ocimum basilicum*	226 ± 2	213 ± 3
*Thymus algeriensis*	125 ± 1	118 ± 1

### Identification of phenolic compounds by HPLC

In this study, 15 phenolic compounds in the ethanolic extracts of *O. basilicum* and *T. algeriensis* were investigated by HPLC*.* The concentrations of the identified polyphenolic compounds in all analysed samples are presented in Table 2 (shown in the order of their retention time). The data in Table 2 revealed that the ethanolic extract of *O. basilicum* had high amounts of rutin, epicatechin and vanillic acid: 476.28 μg/g, 225.01 μg/g, and 138.24 μg/g, respectively. Two phenolic compounds, quercetin (0.36 μg/g) and caffeic acid (6.48 μg/g), were found in low concentrations. In the *T. algeriensis* ethanolic extract, six compounds were detected in notably high concentrations: epicatechin (major compound; 824.79 μg/g), 2, 5-dihydroxybenzoic acid (778.76 μg/g), ellagic acid (374.58 μg/g), rutin (280.39 μg/g), vanillic acid (182.67 μg/g), and naringin (120.67 μg/g). Epicatechin was found to be the major compound in the *Thymus algeriensis* ethanolic extract (824.79 μg/g), and 3, 4-dihydroxybenzoic acid was detected in the lowest concentration (1.42 μg/g). Nonetheless, to the best of our knowledge, HPLC identification of phenolic compounds from *Thymus algeriensis* has never been previously reported.

**Table 2:** HPLC analysis of ethanolic extracts for phenolic compound contents

Phenolic standard compounds	Standard retention time RT (min)	*Ocimum basilicum* (μg/g)	*Thymus* *algeriensis* (μg/g)
Gallic acid	6.8	17.61	10.49
3,4-dihydroxy benzoic acid	10.7	21.56	1.42
4-hydroxy benzoic acid	15.7	46.29	10.03
2,5 dihydroxybenzoic acid	17.2	52.14	778.76
Chlorogenic acid	18.2	25.11	22.68
Vanillic acid	19.2	138.24	182.67
Epicatechin	21.3	225.01	824.79
Caffeic acid	22.7	6.48	33.30
p-coumaric acid	26.1	12.83	83.80
Ferulic acid	30.1	25.00	34.30
Rutin	45.6	476.28	280.39
Ellagic	47.7	27.64	374.58
Naringin	49.7	21.02	120.67
Quercetin	70.4	0.36	2.84
Cinnamic acid	71.1	54.68	20.51

### Essential oil yield and chemical composition

The yields of the essential oils under study were 1.05 and 0.51% for *O. basilicum* and *T. algeriensis*, respectively. Chromatographic analyses resulted in the identification of 61 compounds, representing 98.3% (*O. basilicum*) and 96.5% (*T. algeriensis*) of the essential oils (Table 3). These compounds were grouped into six chemical classes: monoterpene hydrocarbons, oxygenated monoterpenes, sesquiterpene hydrocarbons, oxygenated sesquiterpenes, apocarotenes, and non-terpene derivatives.

**Table 3:** Chemical composition of the essential oils extracted from the leaves of *O. basilicum* and *T. algeriensis*

Constituents	LRI	*O. basilicum* *(%)*	*T. algeriensis* *(%)*	Method of identification^b^
α-pinene	941	0.3	6.8	MS; LRI; RC
camphene	955	- ^a^	0.8	MS; LRI; RC
sabinene	977	0.4	0.7	MS; LRI; RC
β-pinene	982	0.6	1.2	MS; LRI; RC
myrcene	993	1.3	1.2	MS; LRI; RC
3-octanol	994	-^a^	- ^a^	MS; LRI; RC
α-terpinene	1020	- ^a^	- ^a^	MS; LRI; RC
*p*-cymene	1028	- ^a^	0.3	MS; LRI; RC
limonene	1032	- ^a^	2.7	MS; LRI; RC
1,8-cineole	1034	9.2	4.1	MS; LRI; RC
*(Z)*-β-ocimene	1042	0.7	- ^a^	MS; LRI; RC
*(E)*-β-ocimene	1052	0.5	1.5	MS; LRI; RC
γ-terpinene	1063	- ^a^	- ^a^	MS; LRI; RC
*cis*-sabinene hydrate	1070	- ^a^	- ^a^	MS; LRI; RC
terpinolene	1090	0.2	0.4	MS; LRI; RC
linalool	1101	52.1	1.2	MS; LRI; RC
nonanal	1104	- ^a^	- ^a^	MS; LRI; RC
pentylisovalerate	1108	0.1	- ^a^	MS; LRI
1-octen-3-yl acetate	1111	0.1	0.4	MS; LRI; RC
*cis-p*-menth-2-en-1-ol	1123	- ^a^	- ^a^	MS; LRI
3-octyl acetate	1126	0.2	- ^a^	MS; LRI; RC
α-campholenal	1127	- ^a^	0.5	MS; LRI
camphor	1145	- ^a^	1.1	MS; LRI; RC
*trans*-verbenol	1147	- ^a^	0.5	MS; LRI; RC
δ-terpineol	1172	0.2	- ^a^	MS; LRI
4-terpineol	1179	0.1	0.5	MS; LRI; RC
α-terpineol	1191	5.7	4.9	MS; LRI; RC
*cis*-dihydrocarvone	1195	- ^a^	- ^a^	MS; LRI; RC
*trans*-carveol	1219	- ^a^	0.4	MS; LRI; RC
*(Z)*-3-hexenyl isovalerate	1238	- ^a^	- ^a^	MS; LRI; RC
pulegone	1239	0.2	- ^a^	MS; LRI; RC
neral	1242	- ^a^	0.2	MS; LRI; RC
carvone	1244	- ^a^	0.3	MS; LRI; RC
linalyl acetate	1259	19.1	- ^a^	MS; LRI; RC
bornyl acetate	1287	- ^a^	3.1	MS; LRI; RC
dihydroedulan IA	1292	- ^a^	- ^a^	MS; LRI
isodihydrocarvyl acetate	1329	- ^a^	- ^a^	MS; LRI
α-terpinyl acetate	1352	- ^a^	47.4	MS; LRI; RC
*cis*-carvyl acetate	1364	- ^a^	- ^a^	MS; LRI; RC
neryl acetate	1365	1.8	9.6	MS; LRI; RC
geranyl acetate	1383	3.6	0.9	MS; LRI; RC
β-elemene	1392	- ^a^	- ^a^	MS; LRI
*(Z)*-jasmone	1395	- ^a^	- ^a^	MS; LRI; RC
β-caryophyllene	1419	1.0	0.6	MS; LRI; RC
β-copaene	1430	- ^a^	- ^a^	MS; LRI
aromadendrene	1440	- ^a^	- ^a^	MS; LRI; RC
α-humulene	1455	0.1	- ^a^	MS; LRI; RC
*(E)*-β-farnesene	1458	0.1	- ^a^	MS; LRI; RC
*cis*-muurola-4(14), 5-diene	1463	0.1	- ^a^	MS; LRI
germacrene D	1482	- ^a^	0.6	MS; LRI
bicyclogermacrene	1496	- ^a^	–	MS; LRI
germacrene A	1505	- ^a^	- ^a^	MS; LRI
δ-cadinene	1524	- ^a^	- ^a^	MS; LRI
*(E)*-α-bisabolene	1542	- ^a^	0.2	MS; LRI
*(E)*-nerolidol	1564	- ^a^	3.5	MS; LRI
spathulenol	1577	- ^a^	- ^a^	MS; LRI
caryophyllene oxide	1582	0.1	0.9	MS; LRI; RC
viridiflorol	1591	0.4	- ^a^	MS; LRI
1, 10-di-*epi*-cubenol	1615	0.1	- ^a^	MS; LRI
T-cadinol	1641	- ^a^	- ^a^	MS; LRI
T-muurolol	1642	- ^a^	- ^a^	MS; LRI
Monoterpene hydrocarbons		4.0	15.6	
Oxygenated monoterpenes		92.0	74.7	
Sesquiterpene hydrocarbons		1.3	1.4	
Oxygenated sesquiterpenes		0.6	4.4	
Apocarotenes		0.0	0.0	
Non-terpene derivatives		0.4	0.4	
Total identified		98.3	96.5	

^a^: not detected in the sample

^b^method of identification: *MS* Mass spectrum, *LRI* Linear retention index, *RC* Reference compound

In the essential oil of *O. basilicum*, 26 compounds were identified, accounting for 98.3% of the oil. Interestingly, the content of oxygenated monoterpenes (92.0%) was 23 times greater than that of monoterpene hydrocarbons (4.0%). Linalool (52.1%) and linalyl acetate (19.1%) were detected as the major compounds in the oil. The composition of the essential oil of *O. basilicum* is extremely variable because of the existence of several chemotypes, i.e., linalool, methyl chavicol, eugenol, methyl eugenol and neral are the main compounds [26]. The essential oil obtained in the present study can be classified as a linalool-linalyl acetate chemotype, a characteristic chemotype of European basil [26].

In the *T. algeriensis* essential oil, 29 compounds were identified, of which 74.7% were oxygenated monoterpenes and 15.6% were monoterpene hydrocarbons. Among the minor compounds, oxygenated sesquiterpene (4.4%) was characterized. The major compounds in *T. algeriensis* EO were α-terpinyl acetate (47.4%), neryl acetate (9.6%), and α-pinene (6.8%). The main compounds in essential oil from *T. algeriensis* from different geographical areas have been frequently reported to include camphor and/or α-pinene, while thymol and linalool have been occasionally reported as the main compounds [27]. Interestingly, the chemical composition of the *T. algeriensis* essential oil reported in this study appears to be unique, as α-terpinyl acetate and neryl acetate were detected as the major compounds in the oil but thymol was not detected at all.

### Antioxidant activities

#### DPPH assay

DPPH is a stable radical that appears purple in solution and has an absorbance maximum at 515 nm. Upon reduction, its colour changes into yellow with a concomitant decrease in absorbance at 515 nm. The colour change is monitored spectrophotometrically and is utilized for the determination of antioxidant capacity [28, 29]**.** To evaluate the different antioxidant potentials of the phenolic extracts and essential oils, their IC_50_ values, as determined by the DPPH assay, were compared to the IC_50_ of the reference compound ascorbic acid (vitamin C) in mg/mL. The results are summarized in Table 4. The ethanolic extracts demonstrated excellent DPPH radical-scavenging activities. Generally, the activity associated with the *O. basilicum* extract was stronger than that associated with the *T. algeriensis* extract, and both extracts had less activity than ascorbic acid (IC_50_ = 0.002 ± 3.826 × 10^− 6^ mg/mL). For the essential oils, the strongest activity was associated with the *T. algeriensis* oil, with an IC_50_ value of 1.437 mg/mL, followed by BHA (IC_50_ of 1.437 mg/mL) and the *O. basilicum* oil (IC_50_ of 16.296 mg/mL). However, all examined samples were less active than the standard antioxidant ascorbic acid.

**Table 4:** IC_50_ (DPPH), IC_50_ (ABTS), AEAC (FRAP assay), AEAC (Phosphomolybdenum assay) of the extracts, ascorbic acid, and BHA

Plant/Control	Extract	IC_50_ (DPPH) (mg/mL)	IC_50_ (ABTS) (mg/mL)	AEAC-FRAP assay (μg/mL)	AEAC- Phosphomolybdenum assay (mg/mL)
*Ocimum basilicum*	EE	0.679 ± 0.0383^a^	0.970 ± 0.022^a^	3.657 ± 0.009 ^a^	0.005 ± 0.0003^a^
	EO	16.296 ± 0.394^c^	0.6870 ± 0.0203^a^	0.003 ± 0.0007 ^c^	0.760 ± 0.001^c^
*Thymus algeriensis*	EE	1.560 ± 0.010 ^a^	1.743 ± 0.195^a^	0.897 ± 0.064^d^	0.007 ± 0.0006^a^
	EO	1.437 ± 4.51 E-05^d^	0.8960 ± 0.203^a^	1.387 ± 0.265^d^	0.432 ± 0.001^c^
Ascorbic acid		0.002 ± 3.826 E-06^b^	0.001 ± 5.13E-05^b^	ND	ND
Vitamin E		ND	ND	ND	0.674 ± 0.057^b^
Trolox		ND	0.003 ± 3.35E-05^b^	ND	ND
BHA		1.685 ± 0.658^b^	ND	1000 ± 0.047^b^	ND

*EE* Ethanol extract

*EO* Essential oil

*ND* Not determined

Each value in the table is represented as mean ± SD (*n* = 3). In the same column, means not sharing the same letter are significantly different at *P* < 0.05 probability level in each column

#### ABTS assay

The results of this assay are shown in Table 4. Based on the results, the EOs appeared to possess higher anti-free radical activity than the ethanolic extracts. The ethanolic extract of *O. basilicum* demonstrated higher antiradical activity than the ethanolic extract of *T. algeriensis,* which may be attributed to its high phenolic content (rutin, vanillic acid, epicatechin); such compounds are responsible for trapping the cation radical ABTS^•+^ by providing H^+^.

#### Ferric-reducing antioxidant power test

Table 4 shows the AEAC values for the ethanolic extracts and essential oils obtained from the studied plants, together with that of BHA, which was chosen as the standard antioxidant. Considerable attention has been focused on the determination of total antioxidant capacity using the antioxidant activity of ascorbic acid in terms of equivalence and reflected in the AEAC, defined as the concentration of ascorbic acid (μg/mL) that gives an antioxidant power equivalent to that of a concentration of 1 mg/mL of the EO and EE. A high AEAC indicates a notable antioxidant capacity [30]. Relative to that of the positive control, the EE of *O. basilicum* had the most potent activity (IC_50_ of 3.657 ± 0.009 μg/mL), followed by the EO of *T. algeriensis* (IC_50_ of 1.387 ± 0.265 μg/mL). However, the EE of *T. algeriensis* demonstrated a lower antioxidant potential, with an IC_50_ of 0.897 ± 0.064 μg/mL. Notably, the EOs from both plants demonstrated poor Fe^2+^ reducing potential relative to the standard antioxidant BHA.

#### Phosphomolybdenum assay

The different extracts of *O. basilicum* and *T. algeriensis* were also used to determine their antioxidant capacities from the formation of the green phosphomolybdenum complex. The antioxidant activities of the extracts were referenced to that of ascorbic acid in terms of equivalence by calculating the AEAC value. This method is based on the reduction of molybdenum from an oxidation state of VI to an oxidation state of V in the presence of an antioxidant. This reduction is materialized by the formation of a greenish complex detectable in the visible region at 695 nm at an acidic pH [31]. The results indicate that under the applied experimental conditions, the *O. basilicum* and *T. algeriensis* EOs are potent antioxidants, while their phenolic extracts have the lowest potential to reduce molybdenum. The *Ocimum basilicum* oil recorded the highest AEAC, which surpassed vitamin E’s AEAC under the same experimental conditions. This result may be attributed to the chemical composition of the *O. basilicum* oil, which mainly contained 92% oxygenated monoterpenes, with linalool as the predominant compound [32].

### Antimicrobial activity

The ethanolic extracts of *O. basilicum* and *T. algeriensis* as well as their essential oils were evaluated in terms of potential antimicrobial activity against a group of pathogenic microorganisms, comprising gram-positive and gram-negative bacteria and a fungus (Table 5). Superior antimicrobial activity was observed against the gram-positive bacteria examined in this study, while both plant extracts and essential oils demonstrated moderate activities against gram-negative bacteria. Interestingly, the antimicrobial activities observed after essential oil treatment appeared to be generally more potent than those associated with the ethanolic extracts of *O. basilicum* and *T. algeriensis.* As shown in Table 5*,* moderate antifungal activity was associated with both the ethanolic extracts and essential oils from *O. basilicum* and *T. algeriensis* when examined against *Candida glabrata* and *Candida albicans*. Numerous literature studies have provided support for the antimicrobial activities of several of the compounds in the *O. basilicum* and *T. algeriensis* ethanolic extracts and essential oils. For instance, quercetin has been reported as a very efficient antimicrobial compound against several pathogenic gram-positive and gram-negative bacteria [23–25]. In addition, it has been shown that the antibacterial activities of flavonoids, such as quercetin, were effectively enhanced in the presence of rutin, a major component of the ethanolic extracts of both plants, although rutin did not show activity on its own [33].

**Table 5:** Antimicrobial activities of *O. basilicum* and *T. algeriensis* ethanolic extracts and essential oils against eight pathogenic microorganisms expressed as MIC (μg/mL) values

Extract	*S. epidermidis*	*S. aureus*	*B. subtilis*	*E. coli*	*K. pneumoniae*	*p. aeruginosa*	*C. albicans*	*C. glabrata*
*O.basilicum* *EE*	128	128	64	128	256	256	64	128
*T.algeriensis* *EE*	128	64	64	256	256	512	128	128
*O.basilicum* *EO*	32	32	16	64	128	256	32	32
*T.algeriensis* *EO*	32	32	32	64	256	512	64	32
*Ampicillin*	2	2	4	16	64	128	–	–
*Amphotericin B*	–	–	–	–	–	–	2	2

### Anticancer activity

The in vitro antiproliferative effects of the ethanolic extracts and essential oils from *O. basilicum* and *T. algeriensis* were assessed on five human cancer cell lines, namely human breast adenocarcinoma MCF-7 and MDA-MB-231 cell lines, the human adenocarcinoma HeLa cell line, the human prostate cancer PC3 *cell line*, and the human leukaemia K56S cell line. The observed antiproliferative activities on the examined cell lines are presented in Table 6 as LD_50_ values, referring to the concentration at which cancer cell survival was reduced by 50%. The dose-dependent curves obtained by nonlinear regression were used to calculate the LD_50_ values. Doxorubicin, a well-known anticancer agent, was used as a positive control and demonstrated LD_50_ values ranging from 1-20 μg/mL against the different cancer cell lines used in this study. Generally, the essential oils from both plants were much more effective in suppressing cancer cell growth than the ethanolic extracts.

**Table 6:** Antiproliferative activities of *O. basilicum* and *T. algeriensis* ethanolic extracts and essential oils on five human cancer cell lines, exposure time 48 h. LD_50_ (μg/mL) values are shown ±SD

Extract	*MCF-7*	*MDA-MB-231*	*HeLa*	*PC3*	*K562*
*O. basilicum* *EE*	> 10,000	> 10,000	> 10,000	> 10,000	> 10,000
*T. algeriensis* *EE*	> 10,000	> 10,000	> 10,000	> 10,000	> 10,000
*O. basilicum* *EO*	1090 ± 64	1012 ± 44	1052 ± 38	1028 ± 78	929 ± 18
*T. algeriensis* *EO*	647 ± 16	715 ± 22	746 ± 19	1067 ± 96	300 ± 13

The phytochemical constituents of the essential oils under investigation include many of the most active naturally existing anticancer phytochemicals, such as 1, 8-cineole and linalool [23–25]. Similarly, polyphenols, such as quercetin, are often reported to exert anticancer activities against various cancer cell lines [25]. Remarkably, the numerous phytochemical constituents in essential oils and their potency to prevent cancer cell growth at fairly low concentrations may be attributed to the additive or synergistic effects of the different components.

## Conclusion

In summary, the chemical composition of ethanolic extracts and essential oils from *Ocimum basilicum* and *Thymus algeriensis* cultivated in the Algerian Saharan Atlas was reported for the first time in this study. A rich flavonoid content of the EEs of both plants was revealed by HPLC analysis of the phenolic compounds. GC-MS analysis demonstrated that linalool (52.1%) and linalyl acetate (19.1%) are the major compounds in the essential oil from *O. basilicum,* while α-terpinyl acetate (47.4%), neryl acetate (9.6%), and α-pinene (6.8%) were identified as the major compounds in the essential oil from *T. algeriensis*. Additionally, diverse bioactivities of the ethanolic extracts and essential oils from both plants were observed against a range of clinically relevant strains of microorganisms, with enhanced activities against gram-positive bacteria. The essential oils from both plants were much more potent in inhibiting cancer cell growth than the ethanolic extracts. Moderate antioxidant activities were elucidated, suggesting their potential for use in pharmaceutical industries and food production technologies.

## Data Availability

The data that support the findings of this study are available from the corresponding author on reasonable request.

## Abbreviations

A_c_: Absorbance of the control reaction
AEAC: Ascorbic acid equivalent antioxidant capacity
A_S_: Absorbance of the test sample
EE: Ethanolic extract
EO: Essential oil
GAE: Gallic acid equivalents
GC-EIMS: Gas chromatography with electron impact mass spectrometry
LD: Lethal dose
MIC: Minimum inhibitory concentration
RE: Rutin equivalents
